# Seasonal effects of *Searsia lancea, Celtis africana* and concentrate supplementation on weight changes, serum and urine metabolites in South African Nguni goats

**DOI:** 10.1007/s11250-025-04523-3

**Published:** 2025-06-20

**Authors:** France Phiri, Arnold Tapera Kanengoni

**Affiliations:** 1 Department of Agriculture and Animal Health, University of South Africa, Private Bag X6, Florida, 1709 South Africa; 2 Production Animal Studies Department, Faculty of Veterinary Sciences, University of Pretoria, Pvt Bag X04, Onderstepoort, 0110 South Africa; 3 Department of Graduate Studies, University of South Africa, Preller Street, Pretoria, 0003 South Africa

**Keywords:** Browse, Concentrate selectors, Secondary plant metabolites, Urinalysis, Health

## Abstract

This study evaluated the seasonal impact of different dietary supplements on male Nguni goats' health, focusing on serum and urine metabolite concentrations. Twenty male Nguni goats (15 ± 1.6 kg) were arranged in a 2 × 4 (season × diet) factorial design using a randomized complete block arrangement with five goats per treatment. All goats received a basal diet of *Eragrostis* hay supplemented with one of four treatments: T1) *Searsia lancea* (SLA), T2) *Celtis africana* (CAF), T3) a 1:1 combination of SLA and CAF (LAC), or T4) a control diet with concentrate. During the wet season, goats fed CAF or SLA showed higher average daily gain (ADG) and average dry matter intake (ADMI) than those on LAC. Significant season × diet interactions were observed for urobilinogen (URO), urine bilirubin (UBIL), urine protein (UPRO), and urine calcium (UCAL). Goats on the control diet exhibited higher URO and UBIL values (*P* < 0.05) than those on browse forages during both seasons. In the wet season, goats on SLA had higher UPRO (*P* < 0.05) than those on LAC. Across seasons, goats on CAF showed higher blood serum gamma-glutamyltransferase (GGT) than those on control or LAC diets (*P* < 0.05). Diet significantly affected (*P* < 0.05) serum GGT, albumin, alkaline phosphatase, urea nitrogen, creatinine, cholesterol, glucose, and inorganic phosphate levels regardless of season. Urinalysis revealed diet-related kidney dysfunction and imbalanced serum metabolites. The study concluded that CAF and SLA supplementation negatively affected renal and hepatic health in goats, warranting further investigation.

## Introduction

Goats and wild ruminant-concentrate-selectors in captivity risk developing diet-related metabolic disorders and potential mortality when provided with concentrated-based diets lacking sufficient browse material (Amina et al. 2020). While browse is essential, it contains secondary plant metabolites (SPM) that could harm goats'health if consumed inappropriately. Free-ranging ruminants can selectively choose browse with low SPM levels, but captive-managed animals lack this ability to self-regulate their intake. According to the 2022 consensus on South African livestock production, there are 170 million chickens, 21.43 million sheep, 12.2 million cattle, and 5.14 million goats (Stats SA 2022). Although the exact population of Nguni goats in South Africa is unknown, they are vital for smallholder farmers, serving cultural, religious, and economic benefits (Mathapo et al. 2024). Goats have served as model organisms for studying the nutritional challenges faced by captive-managed wild ruminant-concentrate-selectors. The recent surge in research exploring alternative animal feed sources, particularly for goats (Ay et al. 2023), may benefit the management of captive wild ruminant-concentrate-selectors. South Africa has approximately 36,536,940 hectares of browsing land (Stats SA 2022). The diverse vegetation provides goats with access to a variety of browse plants that contain beneficial compounds supporting liver function. Elevated levels of enzymes like alkaline phosphatase and aspartate aminotransferase may indicate liver damage; however, antioxidants found in browse plants can help alleviate this issue (Muir et al. 2008). Additionally, browse can reduce metabolic stress, which is linked to increased levels of glucose, urea nitrogen, and creatinine—important indicators of kidney function (Turner and Belesky 2010). In South Africa's Gauteng province, *Searsia lancea* (*S. lancea*) and *Celtis africana* (*C. africana*) have been identified as suitable browse options due to their availability, despite containing high SPM levels (Phiri et al. 2022). The relationship between SPM consumption and subclinical pathology in ruminant concentrate selectors remains poorly understood, particularly because monitoring subclinical effects presents significant challenges. While urinalysis is a valuable diagnostic tool, its application in monitoring goats'metabolic health has been limited. To address this knowledge gap, we conducted a study comparing the physiological responses of Nguni goats fed different diets: *S. lancea*, *C. africana*, or a 1:1 mixture of both, supplemented with *Eragrostis* hay. The study specifically examined urine and blood metabolite composition in these groups compared to concentrate-fed controls, hypothesizing that SPM consumption would not cause subclinical pathology in ruminant-concentrate-selectors.

## Materials and methods

### Study area

The research was carried out at the Agricultural Research Council-Animal Production (ARC-AP) in Irene, located at 25°89.9′23″ S, 28°21.5′23″ E. The minimum and maximum temperatures in January were 17 °C and 28 °C respectively, while in July, they ranged from 5 °C to 21 °C.

### Animals and their management

Twenty male indigenous Nguni goats, each weighing an average of 15 ± 1.6 kg (mean ± SD), were housed in individual pens measuring 1.5 m in length by 2.5 m in width. The experiment was designed as a 2 × 4 factorial arrangement, considering seasons and diets, and was conducted using a randomized complete block design. Each group consisted of five goats, kept individually and assigned to the treatments. The goats were acquired from smallholder farmers in the Sekhukhune region of Limpopo Province, South Africa where they were free-ranging. They were transported to the ARC–AP, ensuring compliance with animal welfare standards and health measures. The goats were adapted for seven days consuming commercial pellets, and *Eragrostis* hay ad libitum before data collection. All the goats received *Eragrostis* hay as a basal diet and were allotted one of four dietary treatments offered *ad lib*, T1) *S. lancea* (SLA), T2) *C*. *africana* (CAF), T3) a mixture of the two browse species in a ratio 1:1 (LAC), and T4) a control diet (CON) comprising *Medicago sativa*, green apples, butternut squash, spinach, and commercial pellets. The control diet was derived from the formulation used by local zoos to feed captive-managed wild ruminant concentrate selectors. Data was collected for 30 days during wet and dry seasons.

### Preparations of feedstuffs

The CAF and SLA browse forages were collected from the ARC, Vegetable, Industrial, and Medicinal Plants (ARC-VIMP) at Roodeplaat, South Africa (25°61.5′47″ S, 28°36.4′35″ E). The CAF and SLA leaves were randomly collected using a tree lopper once a week during each season in the morning hours (08:00 and 10:00 am). The seasons were divided into wet (October to February) and dry (May to July). The concentrates (Epol lamb and ewe 13® pellets) were purchased from Epol Animal Feeds (Epol Animal Feeds®, Westville, South Africa), *Medicago sativa* was purchased from ARC-VIMP, and the green apples, butternut squash, and spinach were procured weekly from the Tshwane fresh produce retail market.

### Measurements

Goat weights were recorded at the start and end of the study. The average daily gain (ADG) was calculated based on the difference between final and initial weight divided by the number of days in the study. Metabolic body weight was used to compare weights across seasons. Daily dry matter intake (DMI) was calculated from recorded daily feed offered and refused and converted to dry matter basis. Goats were adapted for seven days for urine collection using ventral abdomen-attached plastic bags incorporating the preputial orifice (Jia et al. 2009). Collections were conducted daily between 08:00–11:00. Urine samples were transferred into sealable tubes of 50 mL and kept frozen (−20 °C) in the freezer, until urinalysis. They were tested for urobilinogen (URO), urine bilirubin (UBIL), urine glucose (UGLU), urine protein (UPRO), urine specific gravity (USG), pH, urine blood (UBLD), ascorbic acid (ASC), urine microalbumin (UMA), urine calcium (UCAL), urine creatinine (UCR), and a calculated urine protein creatinine ratio (UPC) using a portable urine analyzer VetScan UA® (Zoetis, Parsippany, New Jersey, USA).

A veterinarian collected blood samples (08:00 to 09:00 am) once at the end of each season. The blood samples were stored in a cooler box with an icepack and transported to IDEXX laboratories for analysis (IDEXX Laboratories Pty Ltd, Kyalami, Johannesburg, South Africa). They were evaluated for General Health Profile (GHP) comprising of serum albumin (SALB), alkaline phosphatase (ALP), alanine amino transaminase (ALT), gamma-glutamyltransferase (GGT), amylase (AMYL), serum urea nitrogen (SUN), serum calcium (SCAL), cholesterol (CHOL), serum creatinine (SCR), globulin (GLOB), serum glucose (SGLU), inorganic phosphate (IPHOS) and serum total protein (STP) using IDEXX Vettest® Chemistry Analyzer (IDEXX Laboratories, Inc., Westbrook, ME. USA).

### Statistical analyses

The final metabolic weight, average daily gain and the blood metabolites (SALB, ALP, ALT, GGT, AMYL, SCR, SUN, SCAL, CHOL, GLOB, SGLU, IPHOS, and STP) per season and urine analytes (URO, UBIL, UGLU, UPRO, USG, pH, UMA, UCAL and UCR) were analyzed using the GLM procedures in Stata (version 17, 2019). The following model was used:

$${y}_{ijk}= \mu + {\tau }_{i}+{\beta }_{j} +{\left(\tau \beta \right)}_{ij}+{\varepsilon }_{ijk}$$

where:

µ = the baseline mean.

τ_i_ = the seasonal effects (dry and wet seasons).

β_j _= the dietary effects (SLA, CAF, LAC and control).

(τβ)_ij_ = the ijth season x diet interaction effects.

ε_ijk_ = the random error of the kth observation from the ij cell.

Significance was set at *p* < 0.05. Means were compared via the adjusted Bonferroni test.

## Results

The average daily gain and metabolic body weight parameters of Nguni goats that were offered *C. africana*, *S. lancea*, and a mixture of LAC forage, along with a control diet, during both wet and dry seasons are presented in Table 1. The results indicate significant interactions between season and diet regarding the final metabolic body weight, average daily gain (ADG), and dry matter intake (DMI) (*P* < 0.05). During the dry season, goats on the control diet exhibited higher final metabolic weight, ADG, and DMI (*P* < 0.05) than those on other diets. Conversely, in the wet season, goats on the browse-supplemented diets showed comparable final metabolic weights, ADG, and DMI to the control group (*P > *0.05). Moreover, DMI was significantly higher during the wet season than in the dry season (*P* < 0.05).

**Table 1:** Body weight parameters of Nguni goats (mean ± sem) offered diets containing control, *Celtis africana* (CAF), *Searsia lancea* (SLA) or *Searsia lancea* and *Celtis africana* mixture (LAC) during the wet and dry seasons

Parameters^2^	Season x Diet								Season
	Wet				Dry				Wet
	CAF	CON	SLA	LAC	CAF	CON	SLA	LAC	
Initial weight kg BW^0.75^	6.4^a^ ± 0.34	6.3^a^ ± 0.34	7.6^ab^ ± 0.34	6.1^a^ ± 0.34	8.7^bc^ ± 0.34	8.7^bc^ ± 0.34	9.5^c^ ± 0.34	8.4^bc^ ± 0.34	6.6^p^ ± 0.20
Final weight kg BW^0.75^	6.8^ab^ ± 0.35	6.6^ab^ ± 0.35	7.7^abc^ ± 0.35	6.1^a^ ± 0.35	8.1^bc^ ± 0.35	10.1^d^ ± 0.35	8.8^ cd^ ± 0.35	7.3^abc^ ± 0.35	6.8^p^ ± 0.25
DMI g/kg BW^0.75^/day	74.6^d^ ± 3.54	62.3^acd^ ± 3.54	56.2^abc^ ± 3.54	63.1^acd^ ± 3.54	52.3^ab^ ± 3.54	73.3^ cd^ ± 3.54	42.5^a^ ± 3.54	49.3^ab^ ± 3.54	64.1^q^ ± 2.70
ADG g/day	13.0^b^ ± 6.09	9.9^b^ ± 6.09	1.7^ab^ ± 6.09	0.36^ab^ ± 6.09	-20.0^ac^ ± 6.09	48.5^d^ ± 6.09	26.0^ac^ ± 6.09	-36.0^c^ ± 6.09	6.2 ± 6.15

Parameters^2^	Season	Diet^1^				*P*-values			
	Dry					Season	Diet	Season x Diet	
		CAF	CON	SLA	LAC				
Initial weight kg BW^0.75^	8.8^q^ ± 0.20	7.5 ± 0.43	7.5 ± 0.43	8.6 ± 0.43	7.2 ± 0.43	0.001	0.152	0.156	
Final weight kg BW^0.75^	8.6^q^ ± 0.25	7.4^xy^ ± 0.41	8.3^y^ ± 0.41	8.2^y^ ± 0.41	6.7^x^ ± 0.41	0.001	0.001	0.001	
DMI g/kg BW^0.75^/day	54.3^p^ ± 2.70	63.4^x^ ± 3.54	67.8^x^ ± 3.54	49.3^y^ ± 3.54	56.2^xy^ ± 3.54	0.001	0.001	0.001	
ADG g/day	-8.5 ± 6.15	-3.6^x^ ± 7.00	29.2^y^ ± 7.00	-2.1^x^ ± 7.00	-17.8^x^ ± 7.00	0.099	0.001	0.001	

Means within a row that do not share a common superscript ^abcd^ for Season x diets effects, ^pq^ for season effects and ^xyz^ for diet effects differ significantly at *P* < 0.05

Parameters^2^; BW^0.75^ = metabolic body weight, ADG = average daily gain, DMI = dry matter intake

Diet^1^; CAF = *Celtis africana*, CON = Control, SLA = *Searsia lancea*, LAC = *Searsia lancea* and *Celtis africana* 1:1 mixture

SEM = standard error of the mean

The urinalysis results displayed in Table 2 also indicate significant interactions between season and diet (*P* < 0.05) for variables such as URO, UBIL, UPRO, USG, pH, and UPC. During the wet season, goats on the control diet had higher URO and UBIL concentrations than those in the browse-supplemented groups (*P* < 0.05). Additionally, control diet goats exhibited higher levels of UPRO, pH, and UPC, along with lower USG values when compared to those on the SLA and LAC diets during the wet season (*P* < 0.05).

**Table 2:** Urinalysis of Nguni goats (mean ± sem) offered diets containing *control, Celtis africana* (CAF)*, Searsia lancea* (SLA) *or Searsia lancea* and *Celtis africana* mixture (LAC) during the wet and dry seasons

Parameters^2^	Season x Diet								Season
	Wet				Dry				Wet
	CAF	CON	SLA	LAC	CAF	CON	SLA	LAC	
URO mg/dL	0.4^a^ ± 0.5	3.5^b^ ± 1.0	0^a^ ± 0.5	0.5^a^ ± 0.5	0.3^a^ ± 0.4	1.0^a^ ± 0.4	1.0^a^ ± 1.0	0^a^ ± 0.5	1.1 ± 0.2
UBIL mg/dL	0.1^a^ ± 0.2	1.6^b^ ± 0.2	0.0^a^ ± 0.2	0.0^a^ ± 0.2	0.2^a^ ± 0.2	0.3^a^ ± 0.2	0.3^a^ ± 0.2	0.0^a^ ± 0.2	0.4 ± 0.1
UPRO mg/dL	220^ab^ ± 51.1	300^b^ ± 57.1	24^a^ ± 51.1	108^a^ ± 57.1	143^ab^ ± 47.0	186^ab^ ± 47.0	158^ab^ ± 57.1	92^ab^ ± 51.1	169 ± 27.1
USG	1.2^abc^ ± 0.0	1.003^a^ ± 0.4	1.2^c^ ± 0.3	1.0^bc^ ± 0.4	1.0^ab^ ± 0.0	1.0^ab^ ± 0.3	1.0^ab^ ± 0.4	1.0^ab^ ± 0.3	1.2 ± 0.2
pH	8.0^abc^ ± 0.4	9.0^c^ ± 0.4	6.9^ab^ ± 0.4	6.8^a^ ± 0.40	8.7^c^ ± 0.3	8.9^c^ ± 0.3	8.5^abc^ ± 0.4	8.6^bc^ ± 0.4	7.7 ± 0.2
UMA mg/L	1.0 ± 0.3	1.0 ± 0.2	0.6 ± 0.2	1.0 ± 0.20	1.0 ± 0.2	0.8 ± 0.2	0.5 ± 0.2	0.6 ± 0.2	1.0 ± 0.1
UCAL mg/dL	12.0 ± 3.0	12.5 ± 3.3	20.0 ± 3.3	22.5 ± 3.4	23.3 ± 3.0	21.7 ± 3.0	20.0 ± 3.3	22.0 ± 3.0	16.4 ± 2.0
UCR mg/dL	110 ± 30.0	113 ± 34.0	150 ± 30.0	150 ± 34.0	100 ± 27.4	125 ± 27.4	88 ± 33.0	100 ± 30.0	129 ± 2.0
UPC	2.8^b^ ± 0.3	3.0^b^ ± 0.3	1.2^a^ ± 0.3	1.5^ab^ ± 0.3	2.5^ab^ ± 0.3	2.3^ab^ ± 0.3	2.3^ab^ ± 0.3	2.0^ab^ ± 0.3	2.2 ± 0.2

Parameters^2^	Season	Diet^1^				*P*-values			
	Dry					Season	Diet	Season x Diet	
		CAF	CON	SLA	LAC				
URO mg/dL	0.6 ± 0.2	0.4^x^ ± 0.3	2.2^y^ ± 0.3	0.5^x^ ± 0.4	0.2^x^ ± 0.4	0.10	0.00	0.00	
UBIL mg/dL	0.2 ± 0.1	0.1^x^ ± 0.1	0.9^y^ ± 0.1	0.1^x^ ± 0.2	0.0^x^ ± 0.2	0.01	0.00	0.00	
UPRO mg/dL	146 ± 25.1	179 ± 34.3	239 ± 36.4	96 ± 38.7	99 ± 38.1	0.6	0.021	0.12	
USG	1.0 ± 0.2	1.2^xy^ ± 0.2	1.1^x^ ± 0.2	1.2^y^ ± 0.3	1.2^xy^ ± 0.3	0.01	0.01	0.00	
pH	8.7 ± 0.2	8.4^xy^ ± 0.2	9.0^y^ ± 0.3	7.8^x^ ± 0.3	7.7^x^ ± 0.3	0.00	0.00	0.05	
UMA mg/L	1.0 ± 0.1	1.0 ± 0.12	1.1 ± 0.3	0.55 ± 0.4	0.67 ± 0.1	0.43	0.07	0.97	
UCAL mg/dL	21.8 ± 1.4	18.1 ± 2.0	17.4 ± 2.1	20.0 ± 2.2	22.2 ± 2.2	0.03	0.32	0.12	
UCR mg/dL	104 ± 14.7	105 ± 20.2	119 ± 21.4	116 ± 22.8	123 ± 22.3	0.22	0.92	0.60	
UPC	2.3 ± 0.2	2.6^y^ ± 0.2	2.6^xy^ ± 0.2	1.8^x^ ± 0.2	1.8^x^ ± 0.2	0.51	0.00	0.04	

Means within a row that do not share a common superscript ^abcd^ for Season x diets effects, ^pq^ for season effects and ^xyz^ for diet effects differ significantly at *P* < 0.05.

Diet^1^; CAF = *Celtis africana*, CON = Control, SLA = *Searsia lancea*, LAC = *Searsia lancea* and *Celtis africana* 1:1 mixture.

Parameters^2^; URO = urobilinogen, UBIL = urine bilirubin, UPRO = urine protein, USG = urine specific gravity, pH = acidity and alkaline, UMA = urine microalbumin, UCAL = urine calcium, UCR = urine creatinine, UPC = Urine protein to creatinine ratio. SEM = standard error of the mean,

The blood metabolites results presented in Table 3 revealed significant interactions between season and diet (*P* < 0.05) for various metabolites, including GGT, SUN, SCAL, CHOL, SGLU, and Total bilirubin. Animals in the control group showed higher concentrations (*P* < 0.05) of GGT and SGLU during the dry season, as well as SUN levels across seasons, compared to the browse-supplemented groups. In contrast, CHOL levels during the dry season were lower (*P* < 0.05) in control animals than in those receiving CAF or LAC. Browse-supplemented animals maintained similar SCAL levels across both seasons (*P > *0.05), while control animals had higher SCAL values in the dry season compared to the wet season (*P* < 0.05). Total bilirubin values were significantly higher (*P* < 0.05) in the wet season than in the dry season for all diets. Serum albumin levels were also higher (*P* < 0.05) in goats receiving CAF and LAC compared to those in the control and SLA groups. Goats on CAF and SLA diets exhibited lower ALP values (*P* < 0.05) than those on the control diet. In contrast, browse-supplemented goats had higher SCR levels (*P* < 0.05) compared to those on the control diet, and control goats had higher IPHOS levels (*P* < 0.05) than the browse-supplemented goats.

**Table 3:** Blood metabolite composition of Nguni goats offered diets containing control*, Celtis africana* (CAF), *Searsia lancea* (SLA) or *Searsia lancea* and *Celtis africana* mixture (LAC) during the wet and dry seasons

Parameters^2^	Season x Diet								Season
		Wet				Dry			Wet
	CAF	CON	SLA	LAC	CAF	CON	SLA	LAC	
GGT U/l	43^ab^ ± 4.1	40^ab^ ± 4.1	36^ab^ ± 4.1	43^a^ ± 4.1	17^c^ ± 4.1	44^a^ ± 4.1	25^bc^ ± 4.1	28^abc^ ± 4.1	40^p^ ± 2.0
STP g/l	70 ± 2.4	66 ± 2.4	64 ± 2.4	66 ± 2.4	68 ± 2.4	65 ± 2.4	66 ± 2.4	69 ± 2.4	66 ± 1.2
ALB g/l	35^abc^ ± 1.0	32^ab^ ± 1.0	31^a^ ± 1.0	34^abc^ ± 1.0	36^bc^ ± 1.0	33^ab^ ± 1.0	34^abc^ ± 1.0	37^c^ ± 1.0	33 ± 0.5
GLOB g/l	35 ± 2.1	34 ± 2.1	33 ± 2.1	32 ± 2.1	32 ± 2.1	32 ± 2.1	32 ± 2.1	32 ± 2.1	34 ± 1.0
ALT U/l	25 ± 4.1	16 ± 4.1	23 ± 4.1	20 ± 4.1	22 ± 4.1	17 ± 4.1	20 ± 4.1	20 ± 4.1	21 ± 2.0
ALP U/l	130^a^ ± 154.1	564^ab^ ± 154.1	157^a^ ± 154.1	534^ab^ ± 154.1	129^a^ ± 154.1	848^b^ ± 154.1	201^ab^ ± 154.1	371^ab^ ± 154.1	346 ± 77.1
SUN mmol/l	6.1^bc^ ± 0.4	9.1^d^ ± 0.4	4.3^b^ ± 0.41	5.1^bc^ ± 0.4	1.5^a^ ± 0.4	6.5^c^ ± 0.4	1.4^a^ ± 0.4	2.1^a^ ± 0.4	6.2^q^ ± 0.2
SCR mmol/l	43^ab^ ± 3.4	30^a^ ± 3.4	41^ab^ ± 3.4	36^ab^ ± 3.4	65^c^ ± 3.4	45^ab^ ± 3.4	68^c^ ± 3.4	59^bc^ ± 3.4	37 ± 1.7
SCAL mmol/l	2.4^abc^ ± 0.1	2.3^c^ ± 0.1	2.3^ac^ ± 0.1	2.4^abc^ ± 0.1	2.5^ab^ ± 0.1	2.6^a^ ± 0.1	2.5^ab^ ± 0.1	2.6^a^ ± 0.2	2.4 ± 0.0
CHOL mmol/l	1.4^a^ ± 0.2	1.2^a^ ± 0.2	1.2^a^ ± 0.2	1.6^ab^ ± 0.2	2.8^ cd^ ± 0.2	1.5^a^ ± 0.2	2.1^bc^ ± 0.2	3.0^d^ ± 0.2	1.3^p^ ± 01
SGLU mmol/l	2.8^a^ ± 0.1	2.9^a^ ± 0.1	3.1^a^ ± 0.1	2.8^a^ ± 0.1	3.0^a^ ± 0.1	4.0^b^ ± 0.1	2.9^a^ ± 0.1	2.7^a^ ± 0.1	2.9 ± 0.1
IPHOS mmol/l	1.6^a^ ± 0.2	2.8^b^ ± 0.2	1.6^a^ ± 0.2	1.5^a^ ± 0.2	1.3^a^ ± 0.2	2.8^b^ ± 0.2	1.9^ab^ ± 0.2	1.5^a^ ± 0.2	1.9 ± 0.1
TotalBil mmol/l	3.0^b^ ± 0.3	2.8^bc^ ± 0.3	3.3^b^ ± 0.3	3.0^b^ ± 0.3	1.0^a^ ± 0.3	1.5^ac^ ± 0.3	1.3^a^ ± 0.3	1.6^ac^ ± 0.3	3.0^q^ ± 0.2
AMYL U/l	13.5 ± 3.3	11.3 ± 3.3	11.5 ± 3.3	11.5 ± 3.3	15.3 ± 3.3	15.5 ± 3.3	10.8 ± 3.3	14 ± 3.3	11.9 ± 2.3

Parameters^2^	Season	Diet^1^				*P*-values			
	Dry					Season	Diet	Season x Diet	
		CAF	CON	SLA	LAC				
GGT U/l	28^q^ ± 2.0	30^x^ ± 2.9	42^y^ ± 2.9	30^x^ ± 2.9	35^xy^ ± 2.9	0.00	0.02	0.00	
STP g/l	67 ± 1.2	69 ± 1.7	65 ± 1.7	65 ± 1.7	68 ± 1.7	0.74	0.36	0.62	
ALB g/l	35 ± 0.5	35^y^ ± 0.7	32^x^ ± 0.7	33^x^ ± 0.7	36^y^ ± 0.7	0.38	0.02	0.33	
GLOB g/l	32 ± 1.0	34 ± 1.5	33 ± 1.5	33 ± 1.5	32 ± 1.5	0.30	0.89	0.86	
ALT U/l	20 ± 2.0	24 ± 2.9	16 ± 2.9	21 ± 2.9	20 ± 2.9	0.62	0.37	0.69	
ALP U/l	387 ± 77.1	129^x^ ± 109.0	706^y^ ± 109.0	179^x^ ± 109.0	453^xy^ ± 109.0	1.00	0.00	0.36	
SUN mmol/l	2.9^p^ ± 0.2	3.8^x^ ± 0.3	7.8^y^ ± 0.3	2.8^x^ ± 0.3	3.6^x^ ± 0.3	0.00	0.00	0.01	
SCR mmol/l	59 ± 1.7	54^y^ ± 2.4	37^x^ ± 2.4	55^y^ ± 2.4	47^y^ ± 2.4	0.00	0.00	0.42	
SCAL mmol/l	2.6 ± 0.0	2.5 ± 0.0	2.4 ± 0.0	2.4 ± 0.0	2.5 ± 0.0	0.11	0.46	0.04	
CHOL mmol/l	2.3^q^ ± 0.1	2.1^y^ ± 0.1	1.3^x^ ± 0.1	1.6^x^ ± 0.1	2.3^y^ ± 0.1	0.00	0.00	0.00	
SGLU mmol/l	3.1 ± 0.1	2.9^x^ ± 0.1	3.4^y^ ± 0.1	3.0^x^ ± 0.1	2.8^x^ ± 0.1	0.28	0.00	0.00	
IPHOS mmol/l	1.9 ± 0.1	1.5^x^ ± 0.2	2.8^y^ ± 0.2	1.7^x^ ± 0.2	1.5^x^ ± 0.2	0.42	0.00	0.21	
TotalBil mmol/l	1.3^p^ ± 0.2	2.0 ± 0.2	2.1 ± 0.2	2.3 ± 0.2	2.3 ± 0.2	0.00	0.14	0.00	
AMYL U/l	13.9 ± 1.6	14.4 ± 2.3	13.4 ± 2.3	11.1 ± 2.3	12.8 ± 2.3	0.41	0.79	0.89	

Means within a row that do not share a common superscript ^abcd^ for Season x diets effects, ^pq^ for season effects and ^xyz^ for diet effects differ significantly at the *P* < 0.05^.^. Diet^1^; CAF = *Celtis africana*, CON = Control, SLA = *Searsia lancea*, LAC = *Searsia lancea* and *Celtis africana* 1:1 mixture. Parameters^2^; GGT = gamma-glutamyl transferase, STP = serum total protein, ALB = albumin, GLOB = globulin, ALT = alanine transaminase, ALP = alkaline phosphatase, SUN = serum urea nitrogen, SCR = serum creatinine, SCAL = serum calcium, CHOL = cholesterol; SGLU = serum glucose, IPHOS = inorganic phosphate, TotalBil = total bilirubin, AMYL = Amylase. SEM = standard error of means, CV = coefficient of variation. Normal ranges from IDEXX Laboratories (IDEXX laboratories Pty, Kyalami, Johannesburg, South Africa)

## Discussion

The interactions between seasonal variations and browse intake significantly impacted goat performance, with notable enhancements in DMI, body weight, and ADG observed in goats consuming CAF during the wet season. This suggests that utilizing SLA, CAF, or their combination (LAC) is a viable strategy to meet the maintenance requirements of goats during this period. Urinalysis highlighted significant interactions between season and diet for key parameters. Control animals consistently demonstrated elevated levels of urobilinogen (URO) and urine bilirubin (UBIL) compared to browse-supplemented groups across both seasons. The observed 0.1 mg/dL urine bilirubin level in browse-fed goats may indicate underlying hepatic insufficiency or bile duct obstruction, as supported by previous findings (Cork et al. 2019). Contrary to expectations (Parrah et al. 2013), proteinuria was noted across all treatment groups, particularly pronounced in the control and CAF groups during the wet season. The slightly alkaline urine pH in these groups could disrupt protein structure (Udeh et al. 2021), potentially contributing to the proteinuria observed. Urine creatinine (UCR) levels exceeded reference values (0.5–2.0 mg/dL) across all treatments (Harwood and Muller 2018), suggesting possible renal dysfunction. The increased UPC ratio further highlights a heightened risk of diet-induced renal disease in goats fed the control or CAF diets. Blood metabolite analysis revealed significant season × diet interactions for GGT, SUN, SCAL, CHOL, and SGLU. Notably, lower levels of GGT, ALT, and total bilirubin were recorded, which may suggest dietary imbalances (Al-Bulushi and Al-Hasani 2017). In terms of calcium metabolism, our findings contrast with earlier work by Daniel and Harper (1934). Recent study indicate that browse species contain higher calcium concentrations than grasses (Tefera and Mlambo 2017). Additionally, contemporary research shows that mineral concentrations, including calcium, are generally higher during the rainy season, which contradicts the previously suggested inverse relationship (Salgado-Beltrán et al. 2024). The stability observed in serum calcium levels across seasons in browse-supplemented animals corroborates findings from Medina-Córdova et al. (2014), which indicate that non-legume browse species consistently provide better mineral content. Despite albumin levels slightly exceeding reference ranges (20–30 g/l) (Kaneko et al. 2008), suggesting possible dehydration (Idamokoro et al. 2017), relatively normal STP and albumin levels imply intact protein metabolism despite the indicated proteinuria, underscoring the complexity of protein dynamics in goats consuming browse with various secondary plant metabolites. Control animals showed higher IPHOS and ALP levels, indicating potential alterations in mineral metabolism relative to goats on natural browse. Cholesterol levels were lower than reference values and exhibited significant season × diet interactions, notably increasing during the dry season in CAF and LAC-fed animals, possibly reflecting metabolic adaptations to nutritional stress. Overall, browse supplementation significantly influenced growth performance and metabolic health in Nguni goats, with variations observed by season. While control animals exhibited superior growth performance, particularly in the dry season, browse supplementation had implications for both renal and hepatic function. The consistent proteinuria across all treatments presents a significant finding that challenges established understandings of goat physiology. In conclusion, diets incorporating concentrates, CAF, or SLA may pose risks to renal health in goats, emphasizing the necessity of comprehensive health assessments when considering alternative feeding strategies. Future research should explore the mechanisms underlying the observed proteinuria and evaluate the long-term health implications of these dietary interventions.

## Data Availability

The datasets generated is available from the corresponding author on reasonable request.
